# Effectiveness of Prenatal Counselling on Physical and Psychological Well-Being Among Women With Preeclampsia at a Tertiary Care Hospital in India

**DOI:** 10.7759/cureus.102409

**Published:** 2026-01-27

**Authors:** Sangeetha C, Prasanna Baby

**Affiliations:** 1 Obstetrics and Gynecology, St. Peter's College of Nursing and Research Institute, Hosur, IND; 2 Faculty of Nursing, Sri Ramachandra Institute of Higher Education and Research, Chennai, IND

**Keywords:** blood pressure, fetal outcome, maternal outcome, preeclampsia, prenatal counselling, proteinuria, well-being

## Abstract

Background and aim

Well-being represents being healthy and experiencing positive outcomes. Pregnancy and well-being are often onerous. Women with preeclampsia face challenging events during pregnancy. The purpose of the research is to enable the physical and psychological well-being of women suffering from preeclampsia and to identify the maternal and fetal outcomes after antenatal counselling.

Methods

An experimental design was adopted for the study. Simple randomization was utilized to enroll 180 pregnant women diagnosed with preeclampsia. Finally, for the post-test, 80 pregnant women in the study group and 87 in the control group were included. Prenatal counselling with routine care was provided to the study group. Pregnant women who completed 20 weeks of gestation were assessed for blood pressure, edema, and proteinuria, and the physical well-being scale and WHO Psychological Well-being Index were administered. After implementing scheduled counselling on completion of the third week, the post-test was done. Maternal and fetal outcomes were found from medical records. Both descriptive and inferential statistics were analyzed using the SPSS 23 software.

Results

The mean systolic blood pressure was significantly decreased from pre-test 150.6 mmHg to post-test score 142.8 mmHg in the study group. There was a significant difference identified in the physical and psychological well-being between the groups at p<0.01 and pregnancy outcome in terms of gestational age at delivery and the Apgar score of newborns.

Conclusion

The research findings confirm that counselling during pregnancy improved the well-being of women with preeclampsia.

## Introduction

Pregnancy and motherhood are joyous moments; every woman desires to enjoy this gift from nature [1]. The prenatal period is a time of physiological and psychological preparation for birth and parenthood [1]. Women undergo significant health problems like unforeseen operative delivery, giving birth to a premature baby, or the death of an unborn baby, resulting in stress [2]. Hypertensive disorders are the leading cause of maternal deaths, and preeclampsia and eclampsia are considered near-miss diseases; the word near-miss is used widely to acknowledge the patient has an organ system defect leading to death if left untreated [3]. Preeclampsia is a progressive multisystem disorder characterized as the new onset of elevated blood pressure at least 140/90 mmHg and either proteinuria, tested by urine using a multiple reagent strip, measured from trace to four or more other signs of end organ dysfunction after 20 weeks of gestation among normotensive women before pregnancy [4]. The Preeclampsia Foundation stated that 76,000 pregnant women and 500,000 babies die from these disorders [5]. In India, the rate of preeclampsia is 28%, and the rate of eclampsia is 7.4%-11.3%; this appears to be greater than the global rate [6]. Delivery is the only cure for preeclampsia and early screening during antenatal check-ups prevents complications [7]. Regular prenatal visits were less satisfying for women in underdeveloped countries [8]. Well-being reflects an individual's sense of feeling good and leading life positively [9]. Counselling significantly improves care for preeclampsia by enhancing diet management, ensuring regular hospital visits, and aiding in edema control. It also supports emotional coping, leading to better physical and psychological well-being.

Worldwide, hypertensive disorders of pregnancy cause 14% of maternal deaths [10]. The maternal mortality ratio currently in India is 190-282 per 100,000 live births. In Karnataka, a southern state of India, 144 maternal deaths per 100,000 live births were reported, which was less than the national average. Maternal deaths and complications in preeclampsia are highly attributed to delays in the recognition of sickness and timely transport facilities for treatment [11]. An interventional study on the effect of home-based care for antenatal women with gestational hypertension reported that the nursing care significantly reduced blood pressure and minimized the preeclampsia-related complications [12]. As preeclampsia reaches a state of severity within a short duration, prenatal education and counselling on self-monitoring enable pregnant women to gain self-confidence and control over their disease [13].

The study objectives are: (i) evaluate the effectiveness of prenatal counselling on physical and psychological well-being among women with preeclampsia; (ii) determine the impact of prenatal counselling on maternal and fetal outcomes among women with preeclampsia; and (iii) associate selected background and clinical variables with physical and psychological well-being in both the study and control groups.

## Materials and methods

Quantitative research with an evaluative approach was adopted to arrive at the objectives of the study. The institutional ethics committee of Sri Ramachandra University (IEC-NI/14/JAN/38/06), Chennai, granted approval for the study. The criteria for ethical considerations were based on the Indian Council of Medical Research (ICMR) guidelines for biomedical research involving human beings.

This study is a randomized controlled trial following the Consolidated Standards of Reporting Trials (CONSORT) guidelines. Data collection period was from January 5, 2016 to December 31, 2018. Initially, the screening tests included checking blood pressure (BP) twice in a two- to four-hour interval, protein in urine by dipstick method, and assessment of edema in antenatal women attending the antenatal outpatient department (OPD) of a selected tertiary care hospital. Participants fulfilling the inclusion criteria, such as pregnant women who completed 20-36 weeks of gestational age and had blood pressure of more than 140/90 mmHg with urine protein measures trace to more than 3+ with or without edema, and diagnosed as preeclampsia were included in the study. After obtaining informed consent, the participants were randomly allotted to the study group or the control group. Pregnant women with signs of eclampsia, history of comorbid diseases such as antepartum haemorrhage, multiple pregnancies, and gestational diabetes mellitus were excluded from the study.

The sample size was calculated using the formula:

$Z = \frac{2 S p^2 \left( Z_{1 - \alpha/2} + Z_{1 - \beta} \right)^2}{(\mu_d)^2}$

where $Sp^2 = \frac{S_1^2 + S_2^2}{2}$, S12=standard deviation in the first group, S22=standard deviation in the second group, μd^2^=mean difference between the samples, α=significance level (95%), and (1 - β)=power.

**Figure 1:**
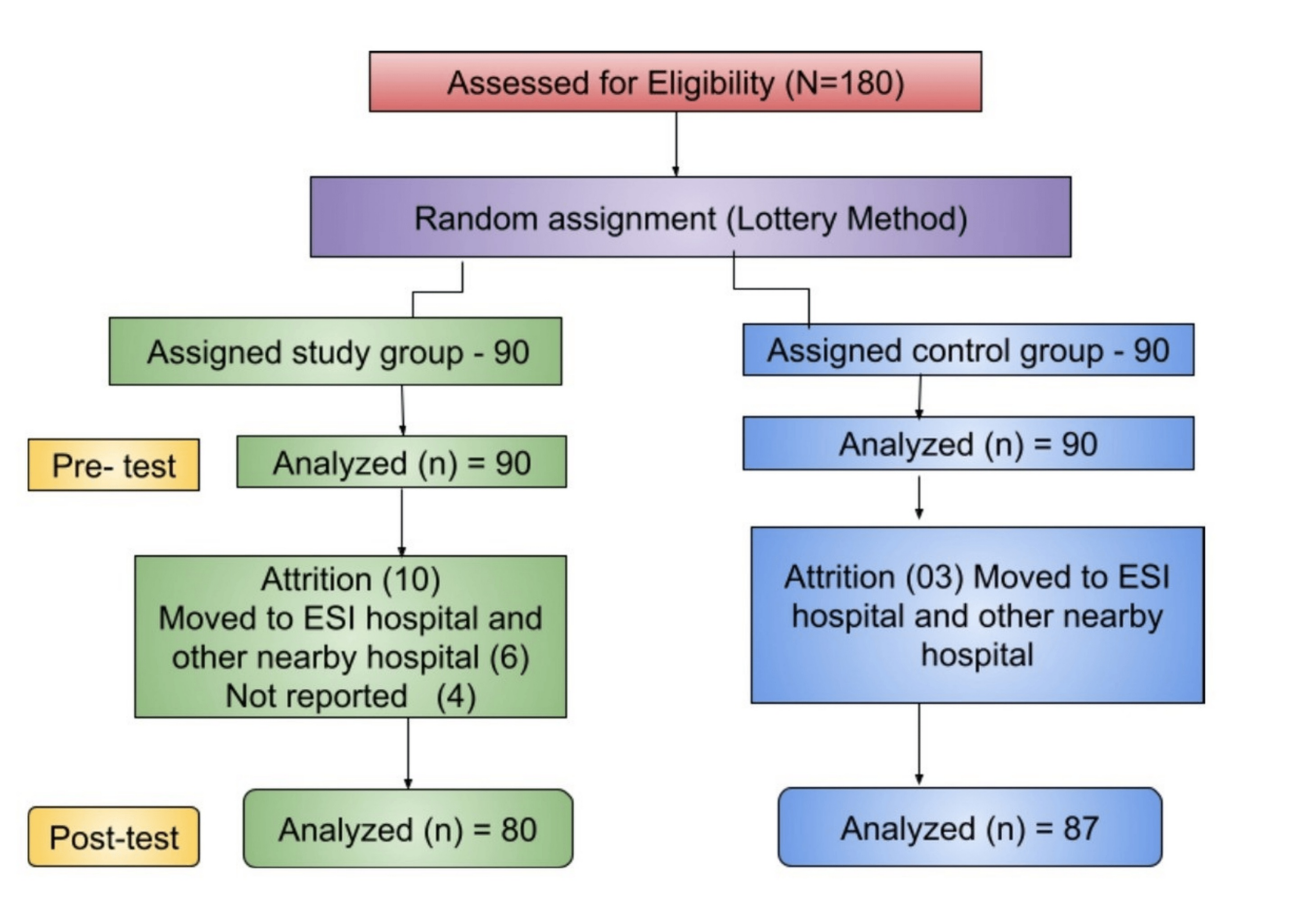
Flow chart of participant recruitment ESI Hospital: Employees' State Insurance Hospital.

The sample size was estimated with 80% power using mean values for S1* and S2* in preeclampsia based on a previous study's mean value of mild preeclampsia (34.5±2.7) and severe preeclampsia (30.4±4.5) [14]. The sample size was determined to be approximately 75 participants in each arm considering a 5% alpha error and 10% drop-out rate. However, as the pilot study revealed significant attrition due to the severity of the condition, the researcher included a 20% increase in the sample size. The final calculated sample size was 180 with 90 participants in each study and control group. Simple randomization (lottery method) was utilized to assign samples to respective groups. There were 180 participants assessed for eligibility and using random allocation, 90 participants were allocated to each group (Figure 1). Among them, in the study group, six participants moved to the ESI Hospital and four did not report for the scheduled counselling process, whereas in the control group, three samples were moved to the Employees' State Insurance (ESI) Hospital.

Description of tool and scoring

The participant's background and clinical variables were obtained by in-person interview. This study includes the physical well-being of women with preeclampsia by assessing blood pressure, edema, proteinuria, and a self-assessment scale.

*Blood Pressure Scoring and Interpretation*: Pre-assessment blood pressure was measured using a standardized aneroid sphygmomanometer. The pregnant women who had blood pressure of 140/90 mmHg and above were considered to have preeclampsia. Scorings less than 139, 140-149, 150-159 and more than 160 mmHg were interpreted as normal, mild, moderate, and severe systolic blood pressure, respectively. Similarly, scorings less than 89, 90-99, 100-109, and more than 110 mmHg were interpreted as normal, mild, moderate, and severe blood pressure, according to the International Society for the Study of Hypertension (2010) recommendations [15].

*Proteinuria Screening*: Proteinuria was assessed using UroColor reagent strips (Abbott, Mumbai, India). Proteinuria scores were interpreted as follows: <1+ scores as absence of preeclampsia; ≥1+ scores as mild preeclampsia; and ≥3+ as severe preeclampsia (American College of Obstetrics and Gynecology) [16].

*Assessment of Edema*: The investigator used the standardized Lippincott pitting edema scale for edema assessment. Pitting edema was graded from trace to severe based on indentation of the edematous skin [17].

The investigator developed a self-assessment scale (see Appendix) consisting of nine items based on the physical experiences of women with preeclampsia on their well-being for the last two weeks. The scale included items such as “I have energy throughout the day without being overly tired", "I feel good about my body", and "I maintain desirable weight gain". The scale, consisting of rarely, sometimes, and most of the time were given the score 0, 1, and 2, respectively. The maximum raw score is 18, a score of 0-8 indicates poor well-being, and 9-18 indicates well-being. The pilot study was conducted among 20 participants who fulfilled the inclusion criteria and the reliability of self-assessment scale obtained was 0.74.

The standardized WHO Well-being Index Scale [18] (see Appendix) was used to assess the psychological well-being of women with preeclampsia. The scale is accepted and has proven mental well-being among pregnancy-related conditions. It consisted of five statements that express the women’s feelings in the previous few weeks. The scoring ranges from 0 to 25, and score below 14-25 indicates well-being and below 13 indicates poor well-being. The reliability of scale was 0.89.

All the instruments were reviewed for content validity by medical and nursing experts, and they were pilot tested to assess their usability and ease of administration.

Women in the study group attended scheduled counselling sessions as given in Table 1. The researcher conducted individual counselling sessions, each lasting for 40-50 minutes. Counselling was done on the following days: on the day of pre-assessment, first counselling, following seventh and 14th day, session II, and session III. Both the control group and study group were given brochures regarding action on preeclampsia during their post-assessment.

**Table 1:** Activities During the Counselling Sessions

Session I	Session II	Session III
On the first day, informative counselling was given on general information such as meaning, causes, investigation, along with dietary guidance, and symptoms of preeclampsia	The second week of the visit: Reinforcement and clarification of the doubts, self-awareness counselling, recognizing the symptoms, rest, exercises, lifestyle modification, and measures to manage the condition	The third week of the visit: Reinforcement and clarification of the doubts from the previous sessions and therapeutic counseling based on individual problem-solving

Both descriptive and inferential statistics were used to analyze the physical and psychological well-being among women with preeclampsia. The statistical software IBM SPSS for Statistics, version 23 (IBM Corp., Armonk, NY) was used to analyze the data at the level of significance, p<0.05. Table 2 depicts the details of the statistical analysis used in this study.

**Table 2:** Details of Statistical Analysis

Study variables	Statistics Analysis
Physical well-being	
(i) Blood pressure	Mean and standard deviation (SD), independent t-test and paired t-test
(ii) Level of edema	Frequency and percentage, chi-square
(iii) Level of proteinuria	Frequency and percentage, chi-square
(iv) Self-assessment scale	Mean and SD, independent t-test and paired t-test
Psychological well-being	Mean and SD, independent t-test and paired t-test
Physical and psychological variables with selected demographic variables	Chi-square, analysis of variance (ANOVA)

Post-assessment was conducted after three consecutive weeks of scheduled counselling on the 21st day of the visit. The maternal and fetal outcome was observed using a checklist from medical records after delivery. Post-assessments of physical well-being were done by assessing blood pressure, edema, proteinuria, and and psychological well-being from self-assessment scale and and WHO Well-being Scale.

## Results

Descriptive analysis of demographic variables showed that 47 (52.2%) participants in the study group and 48 (53.3%) in the control group belonged to the age group of 22-30 years. In the study group, 41 participants (45.5%) were graduates, compared to 26 (28.8%) in the control group. A total of 61 participants (67.7%) from the study group and 58 (64.4%) from the control group belonged to nuclear families. Regarding maternal occupation, 49 participants (54.4%) in the study group and 46 (51.1%) in the control group were employed. Additionally, 58 participants (64.4%) in the study group and 61 (67.7%) in the control group were engaged in sedentary type of work (Table 3).

**Table 3:** Distribution of demographic variables among women with preeclampsia in the study and control group. N=180; study group, n=90, control group, n=90.

Demographic Variables	Study Group		Control Group	
					No.	%	No.	%
	Age (in years)				
15-20 years	2	2.2	1	1.11
21-25 years	8	8.8	12	13.33
26-30 years	47	52.2	48	53.33
31-35 years	25	27.77	26	28.88
>35 years	8	8.8	3	3.33
Educational status				
No formal education	0	0	2	2.22
Primary	15	16.66	21	23.33
High School	14	15.55	14	15.55
Higher Secondary	20	22.22	27	30
Graduate	41	45.55	26	28.88
Type of Family				
Nuclear	61	67.77	58	64.44
Joint	26	28.88	31	34.44
Extended	3	3.33	1	1.11
Family income				
INR 2000- 5000	6	6.66	5	5.55
INR 5001 – 10000	8	8.88	7	7.77
INR 10001-15000	24	26.66	34	37.77
INR 15001-20000	21	23.33	19	21.11
INR >20001	31	34.44	25	27.77
Type of work				
Sedentary	58	64.44	61	67.77
Moderate	26	28.88	23	25.55
Heavy	6	6.66	6	6.66
Place of Residence				
Urban	44	48.88	46	51.1
Semi-urban	36	40	39	43.33
Rural	10	11.1	5	5.55
Maternal Occupation				
Employed	49	54.4	46	51.1
Unemployed	41	45.5	44	48.88

Part I: Comparison of physical well-being in the study and control groups

Table 4 compares the mean score of blood pressure among the participants between the study and control group. The pre-intervention mean systolic and diastolic blood pressure (BP) was similar between the groups. However, post-intervention mean systolic blood pressure and diastolic blood pressure were significantly lower (p<0.01) in the study group compared to the control group.

**Table 4:** Comparison of mean, standard deviation of blood pressure among women with preeclampsia between study and control groups in pre- and post-intervention. S: Significant, NS: non-significant; SD: standard deviation. Pre-intervention study group: n=90, control group, n=90; post-intervention study group: n=80, control group, n=87.  *p<0.01

Blood pressure	Study group		Control group		Independent t-test	p value
	Mean	SD	Mean	SD		
Systolic blood pressure (mmHg)						
Pre-intervention	150.6	11.66	152.03	12.793	0.755	0.451 (NS)
Post-intervention	142.68	18.66	150.67	17.79	2.833	0.005* (S)
Diastolic blood pressure (mmHg)						
Pre-intervention	95.49	9.47	94.16	8.65	0.946	0.346 (NS)
Post-intervention	89.36	10.97	93.76	9.96	2.713	0.007* (S)

Table 5 compares the level of edema between the study group and the control group. The pre-intervention scores of levels of edema showed no appreciable statistical variation between the groups, whereas the post-intervention edema score demonstrated a significant difference between the groups with a p-value of 0.001.

**Table 5:** Comparison of level of edema among women with preeclampsia between study group and control group in pre- and post-intervention S: Significant, NS: non-significant. Pre-intervention study group: n=90, control group n=90; Post-intervention study group n=80, control group, n=87. * p<0.01.

Level of Edema	Study Group		Control Group		χ^2^ value	p value
	No.	%	No.	%		
Pre-intervention						
Absence of edema	44	48.88	32	35.55	3.506	0.173 (NS)
Mild edema	44	48.88	55	61.1		
Severe edema	2	2.2	3	3.33		
Post-Intervention						
Absence of edema	50	62.5	30	34.48	25.126	0.01* (S)
Mild edema	30	37.5	57	65.51		

Table 6 compares proteinuria among women between the groups. During pre-intervention, the level of protein in urine remains similar between the study and control groups. However, the post-intervention score showed a significantly lower level in the study group compared to control group, which exhibited with p-value of 0.01.

**Table 6:** Comparison of level of protein among women with preeclampsia between study and control group in pre- and post-intervention. S: Significant, NS: non-significant. Pre-intervention study group, n=90, control group, n=90; post-intervention study group, n=80, control group, n=87. * p<0.01.

Level of Urine Protein	Study Group		Control Group		χ^2^	p value
	No.	%	No.	%		
Pre-intervention						
Absence of protein	25	27.77	16	17.77	3.146	0.207 (NS)
Mild	52	57.78	62	68.88		
Severe	13	14.44	12	13.33		
Post-intervention						
Absence of protein	36	45	20	22.9	9.45	0.001* (S)
Mild	29	36.25	48	55.1		
Severe	15	18.75	19	21.8		

Table 7 compares the effect of psychological well-being among pregnant women. The pre-intervention mean scores showed no significant difference between the groups, whereas post-intervention, the mean psychological well-being score was significantly higher (p<0.01) in the study group compared to the control group.

**Table 7:** Comparison of frequency and percentage of participant's physical well-being between the study and control groups pre and post intervention S: Significant, NS: non-significant. Pre-intervention study group, n=90, control group n=90. Post-intervention study group, n=80, Post-intervention control group, n=87. *p<0.01

Demographic Variables	Category	Study Group		Control Group			χ²	p value
		No.	%	No.	%			
Pre-intervention	Good well-being	44	48.88	42	45.55		0.020	0.889 (NS)
	Poor well-being	46	51.1	48	53.3			
Post-intervention	Good well-being	57	71.2	39		44.8	11.905	0.001* (S)
	Poor well-being	23	28.8	48		55.2		

**Figure 2:**
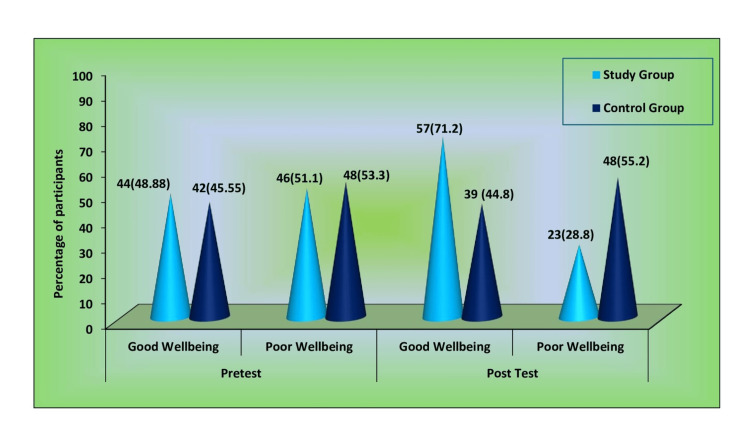
Comparison of frequency and percentage of participant's physical well-being between the study and control groups pre and post intervention Pre-intervention study group, n=90; control group, n=90. Post-intervention study group, n= 80; control group, n=87.

Figure 2 illustrates the comparison of frequency and percentage of participant's physical well-being between study and control group. During the pre-intervention, the participants' physical well-being was similar between the groups. However, post-intervention, most of the participants demonstrated good well-being in the study group than the control group.

Part II: Comparison of psychological well-being in the study and control groups

Table 8 compares the effect of psychological well-being among pregnant women. The pre-intervention mean scores showed no significant difference between the groups, whereas post-intervention, the mean psychological well-being score was significantly higher (p<0.001) in the study group compared to the control group.

**Table 8:** Comparison of mean, SD and post intervention psychological well-being of study and control group among women with preeclampsia. S: Significant, NS: non-significant. Pre-intervention study group, n=90, control group n=90. Post-intervention study group, n=80, post-intervention control group, n=87. *p<0.001

Category	Psychological Well-being				Independent t-test	p-value
	Study Group		Control Group			
	Mean	SD	Mean	SD		
Pre-intervention	12.39	3.931	11.83	3.81	0.934	0.352 (NS)
Post-intervention	15.00	4.36	11.71	4.36	4.677	0.000* (S)

Part III: Comparison of maternal and fetal outcomes in the study and control groups

Table 9 compares post-interventional maternal outcomes between groups among women with preeclampsia. Intrauterine death was significantly lower (p<0.05) in the study group than the control group. The study results proved that gestational age at the time of delivery significantly increased (p<0.01) in the study group compared to the control group. Counselling and continuous monitoring helped the women to extend their gestational age, thereby improving the maternal outcomes.

**Table 9:** Comparison of post interventional maternal outcomes in the study and control group among women with preeclampsia Post-intervention study group n=80, control group n=87. Mode of delivery study group n=69, control group n=64. NVD: Normal vaginal delivery; GA: gestational age; LSCS: lower segmental cesarean section. *p<0.01, **p<0.05.

Maternal Outcome Variables	Category	Study Group		Control Group		χ^2 ^	p-value
		No.	%	No.	%		
Eclampsia	Yes	11	13.75	18	20.68	1.399	0.237 (NS)
	No	69	86.25	69	79.31		
Admission to the intensive care unit (ICU)	Yes	7	8.75	3	3.44	2.081	0.149 (NS)
	No	73	91.5	84	96.55		
Intrauterine death	Yes	11	13.75	23	26.4	4.137	0.042^* ^(S)
	No	69	86.25	64	73.56		
Mode of delivery	NVD	16	39.1	23	71.87	5.0987	0.078 (NS)
	LSCS	53	76.8	41	64.06		
GA at delivery	≤ 37	48	60	73	83.9	11.936	0.001^* ^(S)
	≥38	32	40	14	16.09		

Table 10 depicts the comparison of post-interventional neonatal outcomes between the groups. After the birth, the Apgar score of the newborn showed a statistically significant difference in the study group (p<0.01) compared to the control group.

**Table 10:** Comparison of post-interventional fetal outcome variables between study and control group among women with preeclampsia S: Significant, NS: non-significant. Post-intervention study group, n=69; control group, n=64. *p<0.01. LBW: low birth weight; IUGR: intrauterine growth retardation.

Fetal Outcome Variables	Category	Study Group		Control Group		χ^2^	p value
		No.	%	No.	%		
Preterm baby	≥38	29	42.02	30	46.87	0.861	0.834 (NS)
	33-37	32	46.37	25	39.06		
	29-32	7	10.14	7	10.93		
	≤28	1	1.44	2	2.89		
Apgar score of the newborn	0-3	10	14.49	24	37.5	10.42	0.005* (S)
	4-7	7	10.14	8	12.5		
	7-10	52	75.36	32	50		
Birth weight	Normal	39	56.52	22	34.37	6.753	0.08 (NS)
	Very LBW	3	4.34	3	4.68		
	Extremely LBW	3	4.34	4	6.25		
IUGR	Present	16	20	22	25.2	0.663	0.416 (NS)
	Absent	64	80	65	74.7		

Association between demographic and clinical variables among women with preeclampsia

Table 11 highlights a significant association between post-intervention systolic blood pressure in the study group with family income at a level of significant at p<0.05 and type of work at the p<0.01 level.

**Table 11:** Association between post-intervention systolic blood pressure with selected demographic variables in the study group. n=80; *p< 0.05 ,* p<0.01. SBP: systolic blood pressure; F-value from analysis of variance (ANOVA); S: significant.

Demographic Variables	No.	Mean SBP (mmHg)	SD	F value	p-value
Age in years				0.647	0.58
21-25	6	135	12.247		
26-30	45	144	20.117		
31-35	23	140	17.183		
>35	6	142.5	18.908		
Education status				0.161	0.922
Primary	13	141.54	18.64		
Secondary	11	139.55	14.222		
Higher secondary	17	144.06	19.908		
Graduation	39	143.33	19.748		
Type of family				0.619	0.541
Nuclear	56	142.46	18.42		
Joint	21	144.76	16.619		
Extended	3	132	38.105		
Family income per month				2.59	0.043* (S)
INR 2000-5000	2	170	14.142		
INR 5001- 10000	5	158	17.55		
INR 10001-15000	21	143.1	16.917		
INR 15001-20000	21	142.86	17.647		
INR >20000	31	138.03	18.975		
Type of work				5.004	0.001* (S)
Sedentary	54	146.19	19.246		
Moderate	22	138.18	14.355		
Heavy	4	120	11.547		
Place of residence				0.01	0.99
Urban	40	142.98	19.72		
Semi-urban	32	142.34	18.14		
Rural	8	142.5	17.525		
Maternal occupation				0.578	0.45
Employed	44	144.11	21.063		
Unemployed	36	140.92	15.35		

Table 12 shows a significant association between post-intervention diastolic blood pressure with type of work in the study group at the p<0.05 level.

**Table 12:** Association between post intervention Diastolic blood pressure with selected demographic variables in the study group n=80, *p<0.05; DBP: diastolic blood pressure; F-value obtained from analysis of variance (ANOVA); S: significant.

Demographic Variables	No.	Mean diastolic blood pressure (mmHg)	Standard deviation	F-value	p-value
Age in years				0.107	0.956
21-25	6	88.33	7.528		
26-30	45	89.98	13.178		
31-35	23	88.7	6.255		
>35	6	88.33	11.69		
Education status				0.816	0.489
Primary	13	87.69	12.352		
Secondary	11	86.36	9.244		
Higher secondary	17	88.24	10.146		
Graduation	39	91.26	11.332		
Type of family				0.391	0.678
Nuclear	56	88.73	10.997		
Joint	21	90.48	11.609		
Extended	3	93.33	5.774		
Family income per month				0.673	0.613
INR 2000-5000	2	100	14.142		
INR 5001- 10000	5	92	10.954		
INR 10001-15000	21	98.05	9.437		
INR 15001-20000	21	87.62	10.443		
INR >20001	31	89.65	12.298		
Type of work				4.448	0.015* (S)
Sedentary	54	90.91	11.241		
Moderate	22	88.18	9.069		
Heavy	4	75	5.774		
Place of Residence				0.335	0.716
Urban	40	90.23	11.479		
Semi-urban	32	88.13	10.607		
Rural	8	90	10.69		
Maternal occupation				0.327	0.569
Employed	44	90	12.575		
Unemployed	36	88.58	8.732		

Table 13 shows a significant association between the post-intervention level of edema with the type of family and family income in the control group at the p<0.001 level.

**Table 13:** Association between post-intervention levels of edema with selected demographic variables in the control group. n=87, ** p<0.01, S: significant.

Demographic Variables	Mild	Moderate	Severe	χ^2^ value	p-value
	Number (%)	Number (%)	Number (%)		
Age in years				8.423	0.393
15-20	0	0	1(1.15)		
21-25	0	7(8.05)	4(4.60)		
26-30	2(2.30)	12(13.79)	33(37.93)		
31-35	1(1.15)	8(9.20)	16(18.39)		
>35	0	0	3(3.45)		
Education status				6.32	0.612
No formal education	0	1(1.15)	1(1.15)		
Primary	1(1.15)	2(2.30)	5(5.75)		
Secondary	0	8(9.20)	15(17.24)		
Higher secondary	2(2.30)	6(6.90)	16(18.39)		
Graduation	0	10(11.49)	20(22.29)		
Type of family				28.57	0.001** (S)
Nuclear	1(1.15)	17(19.54)	38(43.68)		
Joint	1(1.15)	10(11.49)	19(21.84)		
Extended	1(1.15)	0	0		
Family income per month				21.14	0.001** (S)
INR 2000-5000	1(1.15)	0	1(1.15)		
INR 5001- 10000	0	4(4.60)	3(3.45)		
INR 10001-15000	1(1.15)	9(10.34)	24(27.59)		
iNR 15001-20000	0	3(3.45)	16(18.39)		
INR >20000	1(1.15)	11(12.64)	13(14.96)		
Type of work				3.85	0.426
Sedentary	3(3.45)	16(18.39)	41(47.13)		
Moderate	0	8(9.20)	14(16.09)		
Heavy	0	3(3.45)	2(2.30)		
Place of Residence				2.35	0.671
Urban	2(2.30)	11(12.64)	32(36.78)		
Semi-urban	1(1.15)	14(16.09)	23(26.44)		
Rural	0	2(2.30)	2(2.30)		
Maternal occupation				0.425	0.808
Employed	1(1.15)	14(16.09)	30(34.48)		
Unemployed	2(2.30)	13(14.94)	27(31.03)		

Table 14 exhibits a significant association between the level of proteinuria with education status in the control group at a p<0.05 level.

**Table 14:** Association between post interventional Proteinuria with selected demographic variables in the control group n=87 *p<0.05, S-significant.

Demographic Variables	Absence	Mild	Moderate	Severe	χ2 value	p-value
	No %	No %	No %	No %		
Age in years					14.25	0.285
15-20	0	0	1(1.15)	0		
21-25	6(6.90)	1(1.15)	2(2.30)	2(2.30)		
26-30	8(9.20)	12(13.79)	19(21.8)	8(9.20)		
31-35	6(6.90)	6(6.90)	5(5.75)	8(9.20)		
>35	0	1(1.15)	1(1.15)	1(1.15)		
Education status					25.51	0.013* (S)
) No formal education	1(1.15)	0	0	1(1.15)		
Primary	2(2.30)	0	3(3.45)	3(3.45)		
Secondary	2(2.30)	12(13.7)	3(3.45)	6(6.90)		
Higher secondary	7(8.05)	5(5.7)	7(8.05)	5(5.75)		
Graduation	7(8.05)	3(3.45)	15(17.24)	4(4.60)		
Type of family					9.20	0.168
Nuclear	10(11.49)	11(12.6)	23(26.4)	12(13.79)		
Joint	9(10.34)	9(10.3)	5(5.75)	7(8.05)		
Extended	1(1.15)	0	0	0		
Family income per month					13.34	0.344
INR 2000-5000	1(1.15)	1(1.15)	0	0		
INR 5001- 10000	2(2.30)	1(1.15)	4(4.60)	0		
INR 10001-15000	7(8.05)	12(13.79)	6(6.90)	9(10.34)		
INR 15001-20000	4(4.60)	3(3.45)	8(9.20)	4(4.60)		
INR >20000	6(6.90)	3(3.45)	10(11.49)	6(6.90)		
Type of work					2.43	0.875
Sedentary	15(17.24)	15(17.24)	19(21.84)	11(12.64)		
Moderate	4(4.60)	4(4.60)	8(9.20)	6(6.90)		
Heavy	1(1.15)	1(1.15)	1(1.15)	2(2.30)		
Place of Residence					2.28	0.892
Urban	11(12.64)	11(12.64)	16(18.39)	7(8.05)		
Semi-urban	8(9.20)	8(9.20)	11(12.64)	11(12.64)		
Rural	1(1.15)	1(1.15)	1(1.15)	1(1.15)		
Maternal occupation					2.59	0.460
Employed	12(13.79)	10(11.49)	16(18.39)	7(8.05)		
Unemployed	8(9.20)	10(11.49)	12(13.79)	12(13.79)		

Table 15 exhibits a significant association between systolic blood pressure with pre-pregnancy BMI, previous history of preeclampsia in the study group at a p<0.01 level.

**Table 15:** Association between post intervention systolic blood pressure with selected clinical variables in the study group. n=80, **p<0.01. SBP: systolic blood pressure; F-value from analysis of variance (ANOVA); S: Significant.

Clinical Variables	No.	Mean SBP (mmHg)	Standard deviation	F-value	p-value
Pre-pregnancy BMI					
Underweight	3	146.7	28.8		
Normal Weight	24	131.6	15.78	6.353	0.001** (S)
Overweight	28	142.6	15.34		
Obese	25	152.8	18.54		
Gestational age at time of recruitment					
≤28 weeks	14	141	13.42		
29-32 weeks	42	138.9	17.09	3.002	0.056
33-36 weeks	24	150.2	22.08		
Family history of preeclampsia					
No	65	142	18.46	0.371	0.544
Yes	15	145.3	19.95		
Previous History of Preeclampsia					
No	66	145.1	18.71	7.061	0.01* (S)
Yes	14	131.1	13.89		
Parity					
1st Pregnancy	45	144.2	17.72	0.436	0.648
2nd Pregnancy	33	141.06	20.42		
3rd Pregnancy	2	135	7.07		
More than 4 pregnancies	0	0	0		

## Discussion

The research findings of the effectiveness of prenatal counselling clearly showed that there is significant improvement in the well-being of women with preeclampsia. The present study results were consistent with a similar study conducted to evaluate the counselling on lifestyle changes in women with preeclampsia. The results revealed that mean systolic blood pressure reduced in the study group from 147.1 mmHg to 142.5 mmHg, and the diastolic blood pressure was decreased from 97.0 mmHg to 90.83 mmHg in the study group (p<0.001) compared to the control group [19N]. The above study results were consistent with a study done on the impact of multi-component lifestyle intervention on hypertensive Iranian women. The post-test mean systolic blood pressure decreased to 153.2±8.1 mmHg from 158.8±6.4 mmHg after four weeks of intervention in the study group. There was a significant change seen in the mean systolic score after six months (p<0.001) [20].

The physiological well-being measures regarding the level of edema and proteinuria revealed that there was a significant difference between the study and control groups at a p< 0.01 level. The above results are consistent with a randomized controlled study conducted on the effect of self-care education and interventions on the physiological status of the feet in pregnant women, which showed that self-care measures reduce edema among pregnant women [21].

The study results showed that prenatal counselling was effective in improving psychological well-being. The above results are consistent with a similar study conducted on the effect of supportive counselling on coping patterns among pregnant women with nausea and vomiting, results showed a significant difference in the study group at a p<0.001 level [22].

In this regard, present study results aligns to randomized controlled trial was conducted to determine the impact of antenatal counselling on parental knowledge and satisfaction without contributing to anxiety. The results revealed that the counselling group had higher knowledge scores (86.3 vs 64.3, p<0.001) and parental satisfaction (p=0.003) [23].

The present study findings demonstrated a significant difference between the groups concerning intrauterine death, gestational age at delivery, and Apgar score at p<05 and p<0.01 levels. The above study findings are analogous to a similar study that assessed the outcomes in pregnant women with preeclampsia in which 77.85% of women having severe preeclampsia were referred to a tertiary care hospital. Among them, there were 62.14% of the patients reported with eclampsia and 17% with intrauterine death. Moreover, 49.28% of women had a caesarean section, 68.57% were preterm, and 47.85% babies were born with low birth weight. And more than half were admitted to the neonatal intensive care unit (NICU) [24].

Another similar study was done to evaluate the efficacy of dietary counselling on the quality of diet, weight gain, and birth weight in mothers with gestational diabetes. The results showed lower weight gain during pregnancy (p=0.062) and higher birth weights of the infants (p=0.047). This supports the present study's maternal and neonatal outcomes [25].

In conclusion, this study's findings show that prenatal counselling leads to a significant improvement in physical and psychological well-being among women with preeclampsia.

Limitations of the study

The inclusion of women with either mild or severe preeclampsia and early or late gestational age could have influenced the outcomes. Counselling schedule was limited to three consecutive weeks because of the inclusion of both mild or severe preeclampsia.

Strengths of the study

Assessment of well-being and conducting prenatal counselling immediately after diagnosing preeclampsia was a challenging process in itself and women showed interest in participating in the study, since it was their felt need. Counselling was challenging since the severity and progress of the disease and the sessions gave timely help to manage the condition.

Recommendations and future directions

A mixed-method study including both qualitative and quantitative measures on the effect of counselling could convey the optimal well-being of women. Self-developed self-assessment scale could be used to assess the well-being during other pregnancy-related conditions. A similar study can be conducted among women with severe preeclampsia and mild preeclampsia separately. A comparative study on preconception and prenatal counselling among women with preeclampsia could be performed. 

## Conclusions

Prenatal counselling for women who are diagnosed with preeclampsia helps to improve their physical and psychological well-being. Counselling enables women to adapt to the changes presented during preeclampsia. Counselling on information and self-awareness related to the disease condition enabled the women to manage the symptoms and improve their well-being. Hence, improving physical and psychological well-being facilitates better maternal outcomes in terms of gestational age at birth, mode of delivery, and intrauterine death, reducing the incidence of maternal complications. The study findings are clinically significant in improving physical and psychological well-being of women with preeclampsia.

## Appendices

**Table 16:** WHO (Five) Wellbeing Index (1998 version) The WHO-5 Well-Being Index is a questionnaire that measures current mental well-being (time frame: the previous two weeks) Accepted and proved the mental wellbeing among pregnancy related conditions. Please indicate for each of the five statements which is closest to how you have been feeling over the last two weeks. Notice that higher numbers mean better well-being. Example: If you have felt cheerful and in good spirits more than half of the time during the last two weeks, put a tick in the box with the number 3 in the upper right corner. Scoring: The raw score is calculated by totaling the figures of the five answers. The raw score ranges from 0 to 25, 0 representing worst possible and 25 representing best possible quality of life.

	Over the last two week	All of the time	Most of the time	More than half of the time	Less than half of the time	Some of the time	At no time
1	I have felt cheerful and in good spirits.						
2	I have felt calm and Relaxed						
3	I have felt active and vigorous						
4	I woke up feeling fresh and rested						
5	My daily life has been filled with things that interest me						

**Table 17:** Self Assessment Scale. Please Read the following and put (√) mark according to your experience in the given space to each sentences. This scale was developed by the authors of this article. *Scoring*: Rarely/if ever, 0; Sometimes, 1; Most of the time, 2. Maximum score 18. A score of 9-18 indicates wellbeing; minimum score 0-8 indicates poor wellbeing. Item nos. 6, 9: Reverse Scoring.

S.NO	Physical Wellbeing	Rarely/ if ever	Some times	Most of the time
1	I maintain a desirable weight gain			
2	I have energy throughout the day without being overly tired			
3	I feel good fetal movements every day.			
4	I do exercises daily.			
5	I feel good about the condition of my body			
6 *	Swelling in the leg, hands, all over the body restricts my activity			
7	I take rest whenever I get time			
8	I get 7-8 hours of sleep each night.			
9. *	Appearance of signs and symptoms makes me weak and powerless.			
